# Effects of Different Oligosaccharides on Growth Performance and Intestinal Function in Broilers

**DOI:** 10.3389/fvets.2022.852545

**Published:** 2022-04-01

**Authors:** Ling Chang, Yanan Ding, Yushi Wang, Zehe Song, Fei Li, Xi He, Haihan Zhang

**Affiliations:** ^1^College of Animal Science and Technology, Hunan Agricultural University, Changsha, China; ^2^Provincial and Ministerial Co-construction of Collaborative Innovation Center for High-quality Animal Products Production, Changsha, China; ^3^Hunan Engineering Research Center of Poultry Production Safety, Changsha, China; ^4^Guangxi Fufeng Agriculture and Animal Husbandry Group Co., Ltd., Nanning, China

**Keywords:** isomalto-oligosaccharide, raffinose oligosaccharide, chitooligosaccharide, gut microbiota, broiler

## Abstract

**Objective:**

This study was conducted to investigate the effects of different oligosaccharides on the growth performance and intestinal function in broilers.

**Methods:**

A total of 360 1-day-old yellow-feather chickens were randomly divided into 5 groups and fed with a basal diet supplemented with 50 mg/kg chlortetracycline (ANT), 3 g/kg isomalto-oligosaccharide (IMO), 3 g/kg raffinose oligosaccharide (RFO), and 30 mg/kg chitooligosaccharide (COS). The experiment lasted for 56 days, with 1–28 days as the starter phase and 29–56 days as the grower phase.

**Results:**

The results showed that dietary supplementation with RFO and COS significantly improved average daily gain (ADG) and average daily feed intake (ADFI) (*p* < 0.05). Relative to the control group, diets supplemented with oligosaccharides dramatically increased the level of serum IgM (RFO, COS), T-SOD (COS), and GSH-Px (IMO and RFO) and the expression of ZO-1(IMO) and claudin-1 (RFO) (*p* < 0.05). Adding antibiotics or oligosaccharides to the diet could remarkedly increase the villus height and villus height (VH)/crypt depth (CD) ratio of each group (*p* < 0.05). Through the ileum α-diversity analysis and comparison of OTU number in each group showed that the microbial richness of the IMO group increased in the starter phase, and that of the RFO and CSO group increased in the grower phase. Additionally, compared with the control group, IMO supplementation increased the level of ileum sIgA (*p* < 0.05) and the content of valeric acid (*p* < 0.05) in the cecum.

**Conclusions:**

In summary, the addition of oligosaccharides in diet can improve the immune function and antioxidant capacity and improve intestinal health of broilers.

## Introduction

An intestinal tract is an important place for digestion and absorption of nutrients in poultry. The intestine plays a dual role that not only maintains the proper permeability to ensure that nutrients are fully digested, but also generates a certain type of cell junction, thus forming the intestinal defense barrier, which is consisted of a mechanical barrier, chemical barrier, immune barrier, and microbial barrier (1). Claudins and occludins are the transmembrane proteins that form the backbone of the intestinal epithelium with tight connections (2). Closed-loop proteins (ZO) are intracellular proteins that provide structural support for tight junctions (3). Mucin secreted by intestinal goblet cells, and mucopolysaccharide, lysozyme, bile salt, and gastric acid secreted by the digestive tract constitute the chemical barrier (4). The microbiota is a stable microecological barrier in the intestine. Meanwhile, gut-associated lymphoid tissue (GALT) and its secreted immune factors form the immune barrier of the intestine, such as cytokines, secretory immunoglobulin A (sIgA) (5). Various barriers are coordinated with each other through different signaling pathways, molecular regulatory mechanisms, and biological functions of organic combination to effectively prevent the invasion of harmful substances through the intestine to poultry.

As feed additives, antibiotics have been widely used in global animal husbandry, which can not only improve the growth performance of animals but also prevent and treat diseases, bringing huge economic benefits to producers (6). These effects are related to the antibiotic function in the gut. In addition, antibiotics serve as an inhibitor to suppress the growth of intestinal bacteria. Bacteria can affect the synthesis of bacterial peptidoglycan, which results in incomplete cell wall components and declined osmotic pressure, which leads to bacterial deformation, rupture, and death (7). However, the excessive use of antibiotics will lead to an imbalance of microecological balance, drug residues in animal products, and antibiotic resistance (8). Thus, it is critical to find alternative substituent products with similar antibiotic effects but without severe contaminations to the environment or public health. Functional oligosaccharides, which are natural, versatile, non-toxic, and non-resistant compounds, are considered as new feed additives that can replace antibiotics environmental-friendly under certain conditions (9). Studies have shown that oligosaccharides could improve animal performance, intestinal microflora, immune regulation, antioxidant, antimicrobial, anti-inflammatory, and cholesterol reduction (10, 11). At present, many studies about the effects of oligosaccharides on animal production are to investigate the consequences of dietary supplementation of a single oligosaccharide (12, 13). There are few studies on the comparison of the effects of various oligosaccharides. In our research, three different kinds of oligosaccharides, isomalto-oligosaccharide (IMO), raffinose oligosaccharide (RFO), and chitooligosaccharide (COS), were added to the basal diet to investigate their effects on growth performance, immune and antioxidant functions, intestinal morphology, and microbiota.

## Materials and Methods

### Animal, Diets, and Experimental Design

The IMO and COS were provided by the Baolingbao Biology Company (Shandong, China). The RFO was provided by Xinrui Biotech (Hunan, China). Based on a single-factor completely randomized design, a total of 360 1-day-old yellow-feather broilers were randomly assigned to five groups with six replicates per treatment and 12 birds per replicate. Five treatments included: (1) CON group: supplied corn-soybean meal basal diet; (2) ANT group: supplied with chlortetracycline (50 mg/kg) based on basal diet; (3) IMO group: supplied with IMO [3 g/kg, according to Thitaram et al. (14) and Zhang et al. (15)] based on basal diet; (4) RFO group: supplied with RFO [3 g/kg, according to Hua et al. (16)] based on basal diet; (5) COS group: supplied with COS [30 mg/kg, according to Khambualai et al. (17)] based on basal diet. The basal diet used in the current study was formulated to meet the recommended nutrient requirements for broilers according to the NRC guidelines [Table 1; National Research Council (18)]. Broilers were reared in a temperature-controlled house under a light schedule of 24 h of light and were provided with mash feed and water *ad libitum*. The whole test period is 56 days, which is divided into two phases: the starter phase (1–28 days) and the grower phase (29–56 days).

**Table 1:** Diet composition and nutrient levels (as-fed basis, %).

**Item**	**Starter phase (1–28 days)**	**Grower phase (29–56 days)**
**Ingredient (%)**		
Corn	65.80	66.30
Wheat bran	0	1.00
Wheat middlings	0.60	0
Soybean meal	25.90	26.90
Fish meal	2.00	0
Soybean oil	1.70	1.80
Vitamin and mineral premix^a^	4.00	4.00
Total^b^	100.00	100.00
**Calculated nutrient level**		
ME (MJ/kg)	12.88	12.32
CP (%)	18.66	17.19
Lys (%)	0.99	0.88
Met (%)	0.27	0.26
Ca (%)	0.19	0.10
Total P (%)	0.42	0.36

a *Premix provided per kilogram of diet: vitamin A, 12,000 IU; vitamin D_3_ 2,500 IU; vitamin E 20 mg; vitamin K_3_ 3 mg; vitamin B_1_ 3.0 mg; vitamin B_2_ 8.0 mg; vitamin B_6_ 7.0 mg; vitamin B_12_ 0.03 mg; pantothenic acid 20.0 mg; nicotinic acid 50.0 mg; biotin 0.1 mg; folic acid 1.5 mg; Fe 96 mg; Cu 25 mg; I 0.9 mg; Zn 98 mg; Mn 105.4 mg; Se 0.3 mg*.

b *These values were calculated from data provided by NRC (18)*.

### Growth Performance

The experiment lasted for 8 weeks. At the beginning of the experiment, the fourth week of the experiment, and the end of the experiment, broilers were weighed by pen (replication), and the feed consumption was recorded by replication, respectively. Average daily feed intake (ADFI), average daily gain (ADG), and feed to gain ratio (F/G) were calculated for periods of 1–28, 29–56, and 1–56 days. In addition, the average body weight on days 28 (D28) and 56 (D56) was calculated.

### Sample Collection

At 28, 56 days of age, after 8 h of starvation, 6 birds (1 bird per replicate) were randomly selected from each treatment group and collected about 5 ml of blood at the venous sinus of the wing vein. The serum, intestinal samples, ileal chyme, and cecal chyme were collected and stored at −80°C for further analysis.

### Determination of Antioxidants and Immunity in the Serum

The serum IgA and IgM were measured using the commercial assay kits purchased from Well Biotech (Jiangsu, China) (19). The levels of MDA, GSH-Px, T-AOC, and T-SOD were using the commercial assay kits purchased from Jiancheng Bioengineering Research Institute (Nanjing, China). The product serial numbers are A003-1-1, A005-1-2, A015-1-2, and A001-1-2, respectively.

### Intestinal Morphology

Briefly, the intestinal samples were dehydrated with increasing concentrations of ethanol, cleared with xylene (Surgipath Medical Industries, Richmond, IL, USA), embedded with paraffin wax (Thermo Fisher Scientific, Kalamazoo, MC, USA), and cut into 4-μm-thick histological sections for hematoxylin and eosin staining. Three straight and well-formed villi were observed for calculating the villous height (VH) and corresponding crypt depth (CD) under the microscope.

### Determination of SIgA Content

Under cold conditions in an ice water bath, the tissue homogenate was prepared using 0.9% NaCl at a weight (g)-to-volume (ml) ratio of 1:9. The homogenate supernatant was obtained by centrifugation (3,500 rpm) for 10 min. The level of sIgA was assayed in the homogenate supernatant of the ileum using the commercial assay kits purchased from Well Biotech (Jiangsu, China).

### Gene Expression

Total RNA of the ileum was isolated using the SteadyPure Universal RNA Extraction Kit (Accurate Bioengineering Co., Ltd., Hunan, China). The mRNA expression levels of mucin-2, claudin-1, occludin, ZO-1, and β-actin were measured by quantitative real-time PCR (RT-qPCR) technique with the primers shown in Table 2. The RT-qPCR was performed using the TB Green Premix Ex Taq (TaKaRa, Biotechnology, Dalian, China). The mRNA levels were calculated using the 2^−Δ*ΔCt*^ method.

**Table 2:** Primer sequence number.

**Gene**	**Accession number**	**Primer sequence (5'-3')**
*β-actin*	L08165	F: GAGAAATTGTGCGTGACATCA
		R: CCTGAACCTCTCATTGCCA
*mucin-2*	XM_421035	F: CTACTTCACCTTCAACCATTACAACG
		R: TCATAGTCACCACCTACTTCTTCAG
*claudin-1*	NM_001012611.2	F: GCAGATCCAGTGCAAGGTGTA
		R: CACTTCATGCCCGTCACAG
*occludin*	NM_205128.1	F: CCGTAACCCCGAGTTGGAT
		R: ATTGAGGCGGTCGTTGATG
*ZO-1*	XM_413773.4	F: GCGCCTCCCTATGAGGAGCA
		R: CAAATCGGGGTTGTGCCGGA

### Determination of Volatile Fatty Acids (VFAs) in the Cecum

The cecum from the frozen tube, thaw, and centrifuge was removed, then, 1 ml of the supernatant was filtered, and the gas chromatography method was used as mentioned in the previous study (20). The content of acetic acid, propionic acid, n-butyric acid, and isobutyric acid was measured by law.

### 16S rRNA Sequencing Analysis

Five samples of cecal contents obtained separately from six random replicates were used to extract DNA for subsequent 16S rRNA sequencing analysis. DNA was extracted from samples of broilers using a Stool DNA Isolation Kit (Tiangen Biotech Co., Ltd., Beijing, China). Amplicons of the V4 hypervariable region of 16S rRNA were amplified using the sample-specific sequence barcoded fusion primers (forward 5'-GTGCCAGCMGCCGCGGTAA-3' and reverse 5'-GGACTACHVGGGTWTCTAAT-3', provided by Allwegene Company, Beijing, China) (21). The volume of PCR reaction was 25 μl, which contains 12.5 μl of Phusion® High-Fidelity PCR Master Mix (New England Biolabs Inc., Beverly, MA,USA), 2 μl of forward and reverse primers, 30 ng of template DNA, and 7.5 μl double distilled H_2_O (ddH_2_O). Cycling parameters were 98°C for 1 min, followed by 30 cycles at 98°C for 10 s, 57°C for 30 s, and 72°C for 30 s, and a final extension at 72°C for 10 min. PCR products were mixed in equidensity ratios and purified with the GeneJET Gel Extraction Kit (Thermo Fisher Scientific Inc., Schwerte, Germany), quantified using real-time PCR, and sequenced at Allwegene Company, Beijing. The sequences were clustered into operational taxonomic units (OTUs) at a similarity level of 97% to generate rarefaction curves (22) and to calculate the richness and diversity indices (23). OTUs representing <0.005% of the population were removed, and taxonomy was assigned by the Ribosomal Database Project (RDP) classifier.

### Statistical Analysis

All statistical analyses were performed using SPSS 20.0 software (SPSS Inc., Chicago, IL, United States). Alpha and beta diversities were analyzed with QIIME (v1.7.0) and displayed with R software (v3.5.1). The differences among groups were compared using one-way ANOVA and Duncan's multiple range test. *p*-values <0.05 were used to indicate statistical significance. β-diversity was assessed by MANOVA and principal coordinate analysis. Significant differences in α-diversity and OTU counts between the different groups were determined by one-way analysis followed by Duncan's multiple comparison test using the SPSS.

## Results

### Growth Performance

As shown in Table 3, in the starter phase (1–28 days), compared with the CON group, the ADG of the RFO group increased by 17.25% (*p* < 0.05), and the ADFI of the RFO group increased by 14.39% (*p* < 0.01). There was no significant difference in growth performance between the experimental groups in the grower phase (29–56 days) (*p* > 0.05). From the whole phase, compared with the CON group, the ADG of the RFO group and ANT group showed an increasing trend (*p* > 0.05). Compared with the IMO group, the ADG of the RFO group increased by 19.44% (*p* < 0.05).

**Table 3:** Effects of different oligosaccharides on growth performance of broilers.

**Items**	**CON Group**	**ANT Group**	**IMO Group**	**RFO Group**	**COS Group**	***p*-value**
Initial body weight (g)	40.08 ± 0.00	41.27 ± 0.00	41.30 ± 0.71	41.50 ± 0.00	40.44 ± 0.00	0.09
D28 body weight (kg)	0.81 ± 0.08^b^	0.90 ± 0.07^a^	0.71 ± 0.06^c^	0.85 ± 0.04^ab^	0.77 ± 0.08^bc^	0.002
D56 body weight (kg)	1.93 ± 0.25^ab^	2.00 ± 0.21^a^	1.75 ± 0.11^b^	2.08 ± 0.10^a^	1.90 ± 0.15^ab^	0.035
**1** **~** **28 d**
ADG(g)	24.52 ± 2.04^b^	26.54 ± 2.73^ab^	23.96 ± 2.11^b^	28.75 ± 1.54^a^	26.21 ± 2.95^ab^	0.013
ADFI(g)	45.38 ± 2.91^bc^	48.20 ± 5.68^abc^	43.70 ± 2.77^c^	51.91 ± 3.36^a^	49.78 ± 3.77^ab^	0.008
F/G	1.85 ± 0.47^c^	1.82 ± 0.07^c^	2.16 ± 0.19^a^	1.94 ± 0.16b^c^	2.06 ± 0.17^ab^	0.001
**29** **~** **56 d**						
ADG(g)	40.12 ± 7.34	40.45 ± 4.93	37.05 ± 2.16	44.12 ± 2.96	40.11 ± 3.19	0.149
ADFI(g)	108.46 ± 14.09	103.70 ± 16.10	99.26 ± 10.93	110.66 ± 11.39	105.15 ± 17.26	0.687
F/G	2.74 ± 0.33	2.56 ± 0.22	2.68 ± 0.21	2.51 ± 0.21	2.65 ± 0.60	0.794
**1** **~** **56 d**						
ADG(g)	33.72 ± 4.50^ab^	35.02 ± 3.73^a^	30.51 ± 2.03^b^	36.44 ± 1.77^a^	33.16 ± 2.61^ab^	0.035
ADFI(g)	76.92 ± 7.87	75.95 ± 7.34	71.48 ± 5.52	81.29 ± 6.68	77.46 ± 9.24	0.282
F/G	2.29 ± 0.17	2.18 ± 0.12	2.35 ± 0.11	2.23 ± 0.15	2.35 ± 0.31	0.440

*ADFI, average daily feed intake; ADG, average daily gain; F/G, feed to gain ratio. Figures with different superscripts within the same column are significantly different (p < 0.05), n = 6. Each mean represents 6 replicates per treatment, with 1 layer per replicate*.

### Immunoglobulin Concentration

As shown in Table 4, at 28 days, compared to the control group, diets supplemented with oligosaccharides could improve the concentration of IgM, and the effect of COS was the most significant (*p* < 0.05). Additionally, compared to the control group, the concentration of sIgA increased in broiler diets supplemented with oligosaccharides (*p* < 0.05) except RFO. At 56 days, IgM levels in the ANT group and RFO group were significantly higher than those in the CON group (*p* < 0.05). There was no significant difference in IgA between the starter and grower phases (*p* > 0.05).

**Table 4:** Effects of different oligosaccharides on serum IgA, serum IgM, and secretory IgA levels in broilers.

**Items**	**CON group**	**ANT group**	**IMO group**	**RFO group**	**COS group**	***p*-value**
28d	IgA (mg/ml)	2.29 ± 0.74	2.17 ± 0.17	1.78 ± 0.14	1.86 ± 0.79	1.94 ± 0.39	0.632
	IgM (mg/ml)	2.33 ± 0.37^b^	2.75 ± 0.79^b^	3.79 ± 1.25^ab^	3.78 ± 0.12^ab^	4.40 ± 0.28^a^	0.047
	sIgA (μg/ml)	0.17 ± 0.01^c^	0.17 ± 0.05^c^	0.47 ± 0.12^a^	0.25 ± 0.10^bc^	0.33 ± 0.01^ab^	0.002
56d	IgA (mg/ml)	1.48 ± 0.27	1.78 ± 0.34	1.66 ± 0.35	1.49 ± 0.03	1.42 ± 0.39	0.532
	IgM (mg/ml)	2.29 ± 0.89^b^	4.78 ± 1.15^a^	2.71 ± 0.27^b^	4.59 ± 1.95^a^	2.57 ± 0.86^b^	0.023
	sIgA (μg/ml)	0.42 ± 0.07	0.39 ± 0.09	0.32 ± 0.09	0.52 ± 0.18	0.38 ± 0.07	0.296

*IgA, immunoglobulin A; IgM, immunoglobulin M; sIgA, secretory immunoglobulin A. Figures with different superscripts within the same column are significantly different (p < 0.05), n = 6. Each mean represents 6 replicates per treatment, with 1 layer per replicate*.

### Antioxidant Capacity

**Figure 1:**
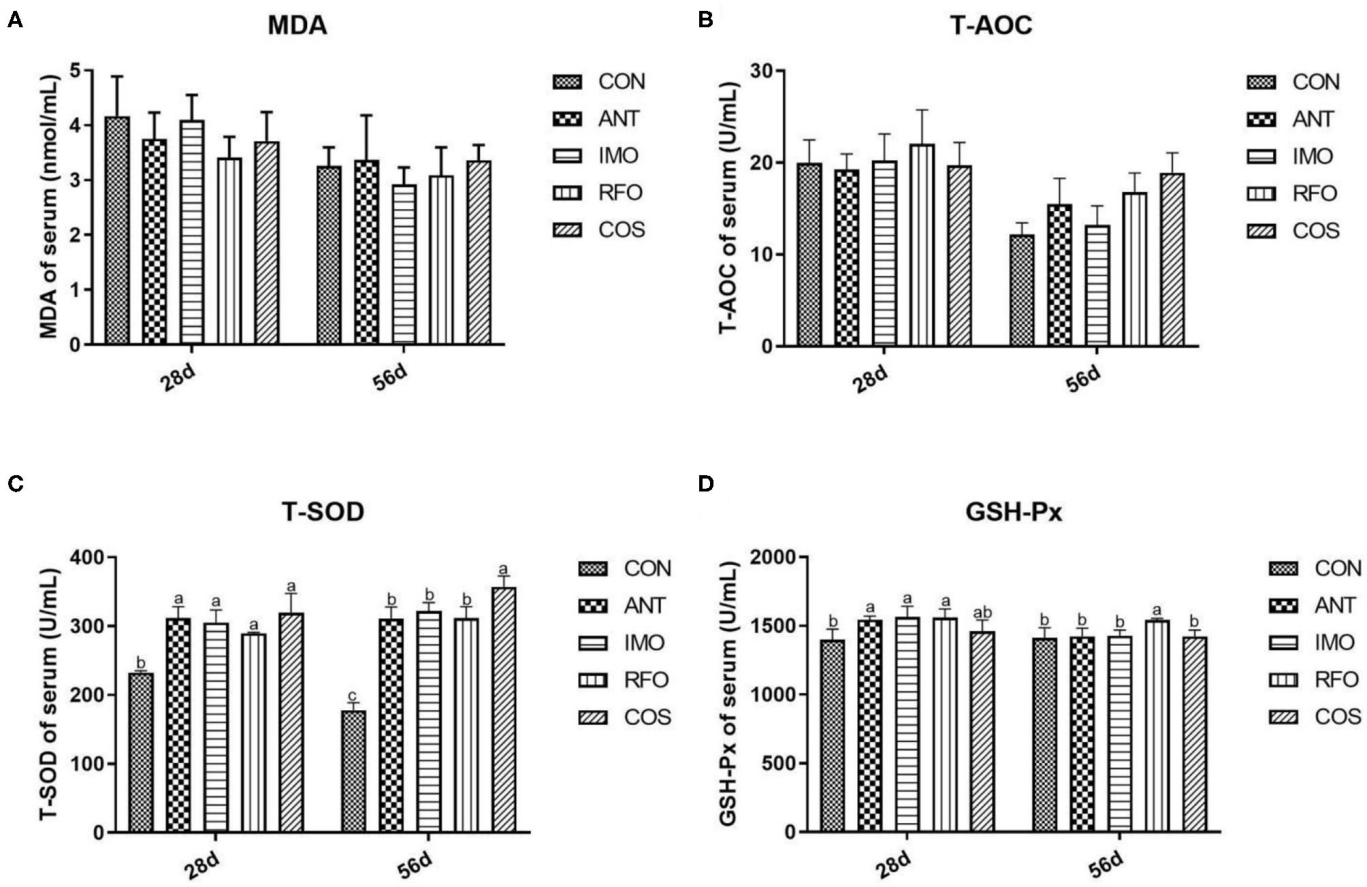
Effects of different treatments on antioxidant indices in serum (*n* = 6). **(A)** MDA, **(B)** T-AOC, **(C)** T-SOD, and **(D)** GSH-Px. Graph bars marked with different letters on top represent statistically significant results (*p* < 0.05) based on ANOVA with Duncan's range tests. CON, control group; ANT, ANT group; IMO, IMO group; RFO, RFO group; COS, COS group.

The antioxidant indices that include the MDA, T-AOC, T-SOD, and GSH-Px content in the serum of broilers are presented in Figure 1. Compared to the control group, dietary supplementation with oligosaccharides, which include IMO, RFO, and COS, remarkably improved the content of T-SOD in the starter and grower phase (*p* < 0.05). Additionally, the RFO group markedly increased the content of GSH-Px in the starter and grower phase, and the IMO group sharply increased the content of GSH-Px in the starter phase compared to the control group. There was no significant difference in MDA levels among groups (*p* > 0.05).

### Intestinal Histomorphology

The normal function and structure of the intestinal tract were indicated by the villus height, CD, and villus length/CD (V/C), as shown in Table 5. In the starter phase, compared with the CON group, the dietary supplementation of oligosaccharides significantly increased the villus height and V/C (*p* < 0.01), and the dietary supplementation of oligosaccharides significantly decreased the CD (*p* < 0.01).In the grower phase, compared with the CON group, the villus height was significantly increased in the RFO group (*p* < 0.01), the CD was significantly decreased in the ANT, IMO, RFO, and COS groups (*p* < 0.01), and the V/C was significantly increased in the ANT, IMO, RFO, and COS groups (*p* < 0.01).

**Table 5:** Effect of different oligosaccharides on ileum morphology of broilers.

**Items**	**CON group**	**ANT group**	**IMO group**	**RFO group**	**COS group**	***p*-value**
28d	Villus height (μm)	639.75 ± 22.90^d^	893.75 ± 16.88^ab^	790.00 ± 36.77^c^	939.33 ± 46.70^a^	880.67 ± 29.50^b^	<0.001
	Crypt depth (μm)	128.33 ± 1.53^b^	151.50 ± 7.78^a^	110.50 ± 4.95^c^	108.33 ± 4.16^c^	106.00 ± 9.90^c^	<0.001
	V/C	4.91 ± 0.05^c^	5.87 ± 0.47^c^	7.16 ± 0.65^b^	8.69 ± 0.67^a^	8.27 ± 0.41^a^	<0.001
56d	Villus height (μm)	701.25 ± 62.66^b^	752.00 ± 26.06^b^	766.50 ± 16.36^b^	835.75 ± 26.80^a^	731.00 ± 7.21^b^	0.005
	Crypt depth (μm)	126.50 ± 4.95^a^	106.75 ± 7.59^b^	104.67 ± 3.79b^c^	94.00 ± 1.41^c^	110.00 ± 4.24^b^	0.004
	V/C	5.62 ± 0.17^d^	6.90 ± 0.54^bc^	7.48 ± 0.11^b^	9.02 ± 0.01^a^	6.66 ± 00.17^c^	<0.001

*Figures with different superscripts within the same column are significantly different (p < 0.05), n = 6. Each mean represents 6 replicates per treatment, with 1 layer per replicate*.

### Intestinal Mucosal Barrier Function

**Figure 2:**
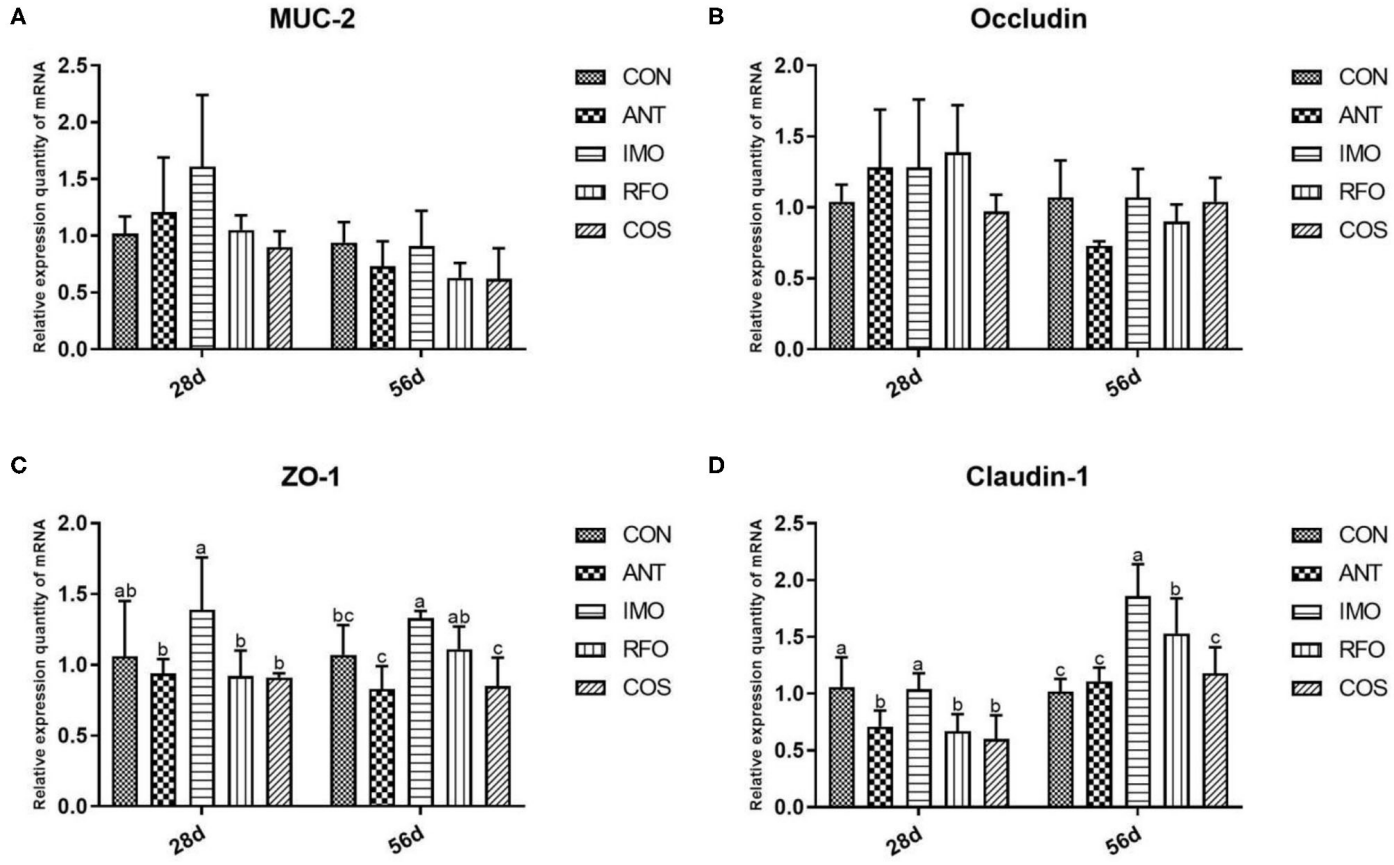
Effects of different treatments on the expression of intestinal related genes and the immune function of ileum in broilers (*n* = 6). **(A)** MUC-2, **(B)** occludin, **(C)** ZO-1, and **(D)** claudin-1. Graph bars marked with different letters on top represent statistically significant results (*p* < 0.05) based on ANOVA with Duncan's range tests. CON, control group; ANT, ANT group; IMO, IMO group; RFO, RFO group; COS, COS group.

The effects of dietary supplementation of oligosaccharides on intestinal mucosal barrier function are presented in Figure 2. Relative to the CON group, the IMO group markedly increased the mRNA expression of ZO-1 and claudin-1 (*p* < 0.05) in the grower phase, but there was no significant difference in the starter phase (*p* > 0.05). RFO group improved the mRNA expression of claudin-1 (*p* < 0.05) in the grower phase compared to the control group.

### Ileum Gut Microbiota

**Figure 3:**
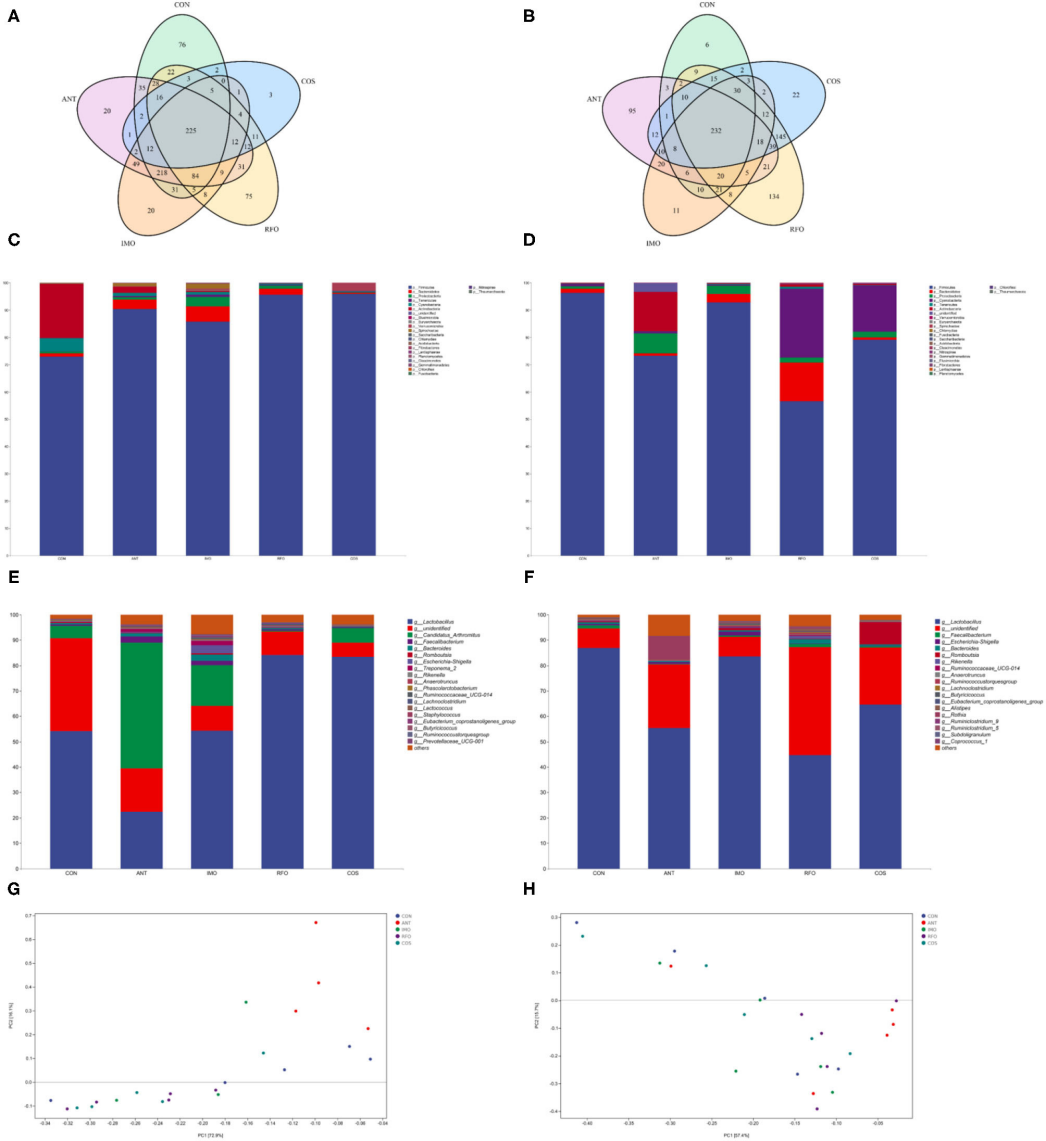
Oligosaccharides alter the composition of the gut microbiota in the broiler (*n* = 5). Venn diagrams showing the unique and shared OTUs in the gut microbiota of the groups **(A,B)**. Community taxonomic composition and abundance distribution map at the phylum level **(C,D)**. Community taxonomic composition and abundance distribution map at the genus level **(E,F)**. PCA clustering analysis **(G,H)**. ANT, IMO, RFO, and COS correspond to supplemented with chlortetracycline, isomalto-oligosaccharide, raffinose oligosaccharide, and chitooligosaccharide in diet, respectively.

Increasing evidence shows that the addition of functional oligosaccharides to diet can affect the structure and composition of gut microbiota (24). Therefore, we sequenced ileum content samples to elucidate the effects of IMO, RFO, and COS on the gut microbiota structure. At the starter phase, 225 OTUs were detected in all groups (Figure 3A). There were 76, 20, 20, 75, and 3 unique OTUs in the CON, ANT, IMO, RFO, and COS groups, respectively. There was no significant difference in the value of Chao1, Goods_coverage, and Shannon between the CON, ANT, and IMO groups. However, significant differences in Chao1, Goods_coverage, and Observed_species were found between broiler in the ANT and COS groups (Supplementary Table 1). At the grower phase, 232 OTUs were detected in all groups (Figure 3B). There were 6, 95, 11, 134, and 22 unique OTUs in the CON, ANT, IMO, RFO, and COS groups, respectively. Significant differences were also found in the Chao1, Goods_coverage measures of the CON and RFO groups (Supplementary Table 2).

The addition of oligosaccharides in diet could reduce the level of *Proteobacteria* in the starter phase and increase the level of *Firmicutes* in broilers. Compared with the CON group, the abundance of *Bacteroidetes, Tenericutes, Euryarchaeota*, and *Spirochaetae* in the IMO group was significantly increased (Figure 3C, Supplementary Table 3). There was no significant difference in gut microbial community abundance among broilers in the grower phase (Figure 3D, Supplementary Table 4). Moreover, the ANT group had significantly reduced *Lactobacillus* and significantly increased *Candidatus_Arthromitus, Faecalibacterium, Bacteroides, Ruminococcaceae_UCG_014*, and *Treponema_2* compared to the CON group in the starter phase (Figure 3E, Supplementary Table 5). Compared with the CON group, supplemented oligosaccharides in the diet could significantly increase the abundance of *Lactobacillus*, except the IMO group. However, IMO and RFO supplementation significantly increased the abundance of *Ruminocaceae_UCG_014* and *Lachnoclostridium* (Figure 3E, Supplementary Table 5). There was no significant difference in species level of gut microbial community abundance among broilers in the grower phase (Figure 3F, Supplementary Table 6). PCA revealed a separation in the microbiota of the groups (Figures 3G,H). Collectively, these results indicated that oligosaccharides could modulate the gut microbiota of the broiler, especially during the starter phase.

### Volatile Fatty Acids

**Figure 4:**
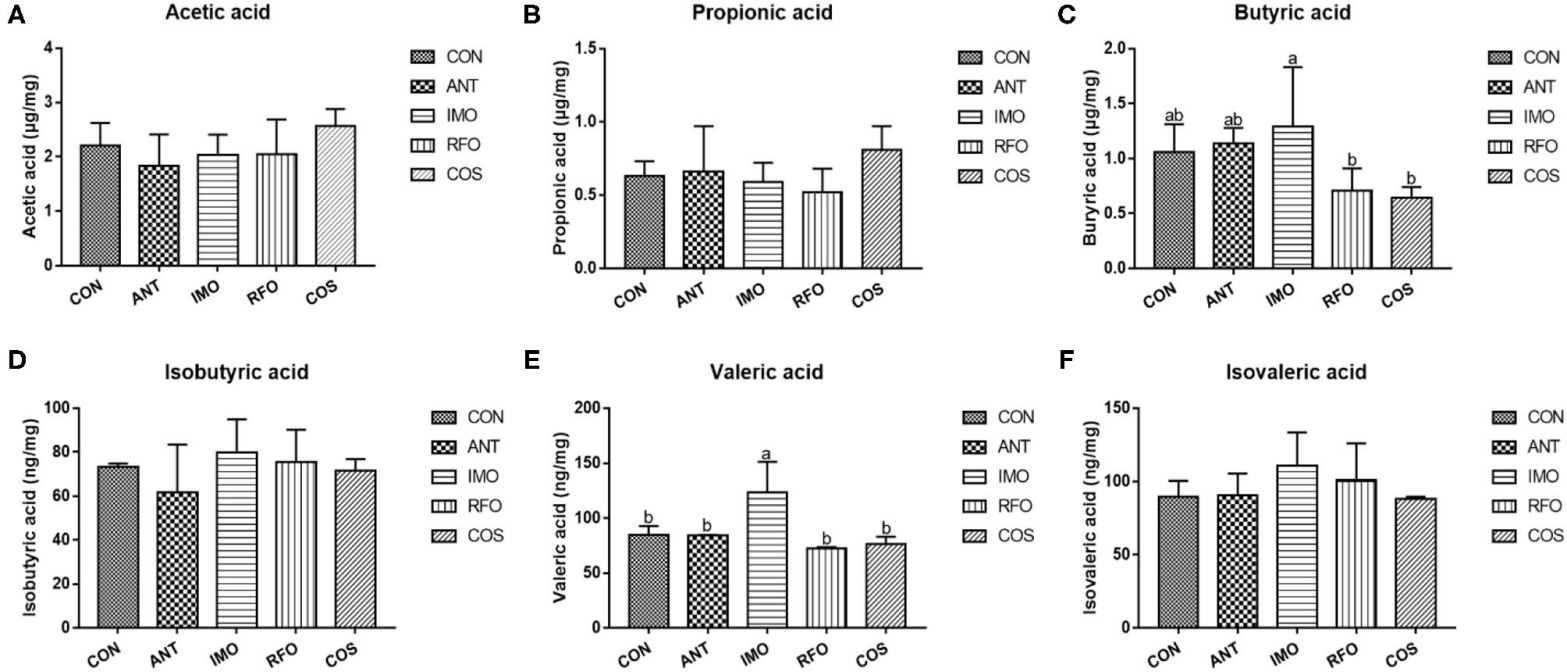
Effects of different oligosaccharides on cecal VFA in yellow-feather broilers (*n* = 6). **(A)** Acetic acid, **(B)** propionic acid, **(C)** butyric acid, **(D)** isobutyric acid, **(E)** valeric acid, and **(F)** isovaleric acid. Graph bars marked with different letters on top represent statistically significant results (*p* < 0.05) based on ANOVA with Duncan's range tests. CON, control group; ANT, ANT group; IMO, IMO group; RFO, RFO group; COS, COS group.

Figure 4 shows that compared with CON and ANT groups, adding IMO in broiler diet can significantly increase the content of valeric acid (*p* < 0.05).

## Discussion

In the post-antibiotics era, prebiotics are proposed as an alternative to antibiotic growth promoters in poultry production. Oligosaccharides can regulate the balance of intestinal flora and build a healthy and stable ecological environment for animals (25–27). Supplemented COS in the diet could improve the production performance, breast meat quality (28), and regulation of intestinal microflora in broilers (29). Small dose IMO (0.1 or 0.2%) could improve the performance of laying hens (30). In this study, the results show that RFO supplementation significantly increased ADG, but decreased F/G in yellow-feather broilers. It is worth mentioning that during the whole rearing period, the RFO group showed improved ADG by 8%, and F/G was significantly decreased by 2.6%, as compared with the control group, even slightly better than the ANT group. Similar to our findings, the previous studies showed that 1.9 mg/embryo RFO significantly increased the bodyweight of broilers (31). These data suggested that RFO could be used as a potential alternative to antibiotics.

The animal immune response is closely associated with immunoglobulins. IgA is associated with mucosal immunity and IgM is correlated with acute infection (32, 33). sIgA plays an important role in intestinal mucosal defense and is the first line of defense on the intestinal surface. sIgA deficiency resulted in bacterial overgrowth, adherence, and translocation (34). IMO was found to play an active role in humoral and cell-mediated immunity for host animals (35). It was reported that the level of IgA in feces was increased when mice were fed a diet supplemented with 20% IMO (36). Meanwhile, 100 mg/kg COS could also promote the development of immune organs in broilers (37, 38). RFO could improve the immunity of the small intestine (39). Similarly, in the current study, 50 mg/kg COS showed improved IgM by 88.8%, and sIgA was significantly increased by 94.1%, as compared with the control group in the starter phase. Simultaneously, IMO and RFO supplementation also had a positive effect on IgM levels. Wu et al. (40) suggested that dietary supplementation of IMO improved the immune function in weaned pigs. Teng and Kim (41) also indicated that oligosaccharides could improve gut development and the immunity of broilers. Our study agreed with the above reports.

GSH-Px and SOD can reduce toxic peroxide and decompose hydrogen peroxide, respectively, eliminate hydroxyl and oxygen free radicals, and protect the cell membrane structure from damage (42). T-AOC is a comprehensive index to reflect the antioxidant activity *in vivo* (43). MDA is the product of membrane peroxidation, and the higher content of MDA would indicate the higher the degree of lipid attacked by reactive oxygen species (44). In our study, the addition of these three oligosaccharides was found to improve the antioxidant capacity of broilers. The previous studies have shown that RFO and IMO supplementation in the diet could significantly improve the antioxidant capacity of yellow-feathered broilers (45, 46). Consistent with the previous studies, supplementation with all three oligosaccharides was found to increased serum T-AOC, T-SOD, and GSH-Px levels in yellow-feather broilers. Noteworthy, chitooligosaccharide was a potential antioxidant and its antioxidant activity was linked to the average molecular weight, the degree of acetylation, and the degree of polymerization (47). In this study, COS with a low degree of polymerization was selected, but the effect was also the most remarkable. It supported the hypothesis that oligosaccharides had effects to improve the antioxidant ability of broilers.

The increase in villus height means the increase in the contact area of nutrients to the intestinal epithelium. The differentiation ability of intestinal epithelial cells depends on the depth of the crypt, which determines the numbers of the intestinal stem cells. When the depth of the crypt is over than the normal CD, it may indicate intestinal mucosal lesions (48, 49). It has been found that the villus height and VH/CD value of broilers increased in a dose-dependent manner with RFO after treatment of fertilized eggs with different doses of RFO, but did not affect the depth of the crypt after 21 days (39). In this research, supplementation with IMO, RFO, and COS significantly increased villus height and VH/CD ratio and decreased CD in the ileum. These data might be owned to the change of intestinal microbiota, which contribute greatly to changes in intestinal morphology when the dietary was supplemented with oligosaccharides.

Volatile fatty acids were one of the most important end products of indigestible foods such as carbohydrates fermented by intestinal microorganisms (50). The fermented short-chain fatty acids can provide energy for intestinal epithelial cells (51). Moreover, intestinal microorganisms can influence the barrier function of the host intestine and regulate intestinal immune response, even systematic immune response (52). Therefore, the logistic characteristics of microbiota can affect the content and composition of volatile fatty acids and the expression patterns of intestine-related genes (53). In our study, PCA of the five treatments showed significant differences in principle components, and three oligosaccharides improved the microbial abundance in the ileum. The previous studies had confirmed that COS had obvious antimicrobial activity, while the intestinal microbiota of broiler chickens was relatively simple structure and low abundance (54). The antimicrobial activity of COS was reflected in its ability to inhibit the growth of pathogens but also slightly inhibit the development of beneficial bacteria (55, 56). However, there is an absence of research on the antimicrobial activity of RFO. According to the differences in performance during the starter and grower phase, RFO was similar to the COS group. The content of butyric acid in the IMO group was significantly greater than that in the RFO and COS groups, and the content of valeric acid was the highest.

**Figure 5:**
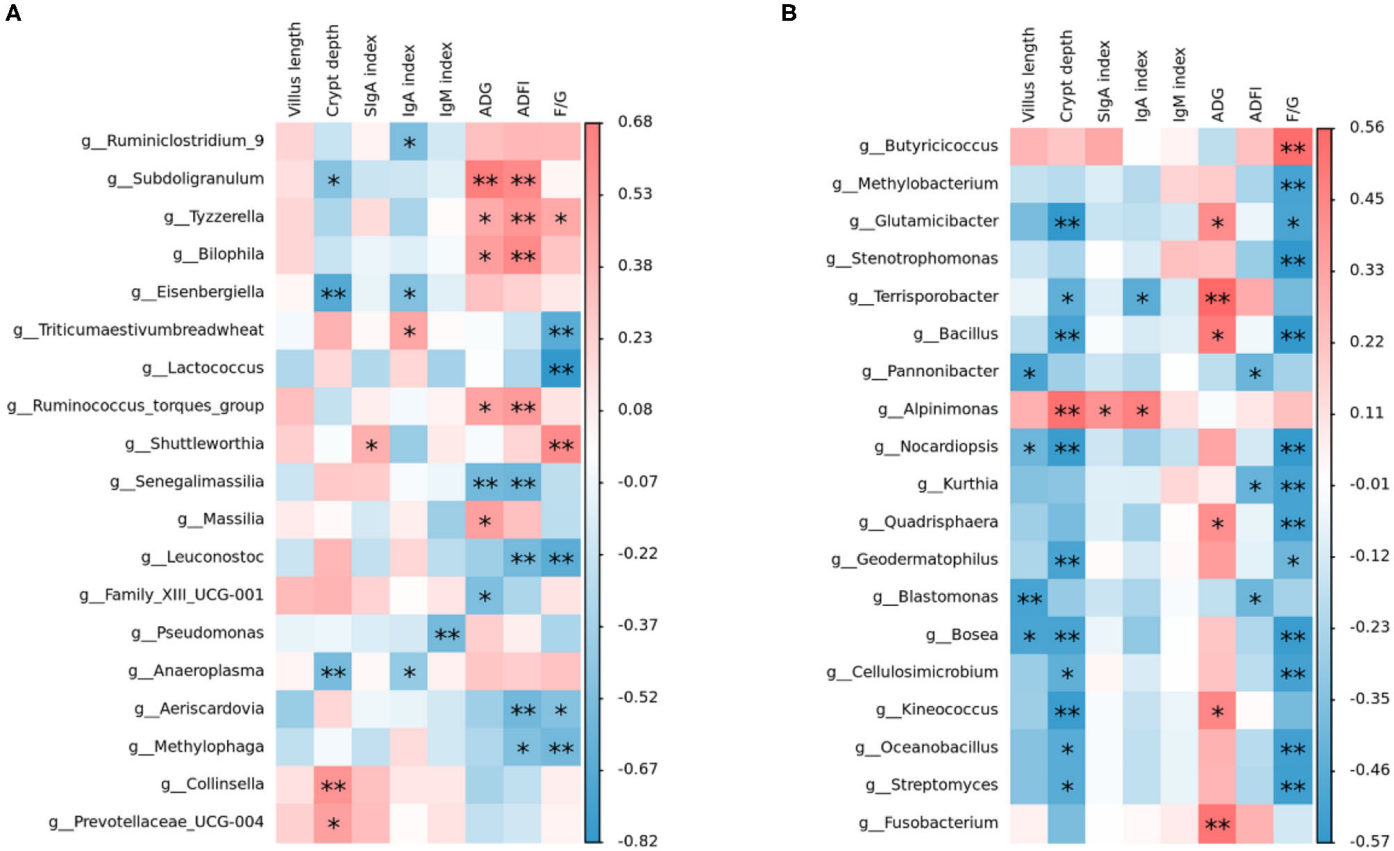
Relationship between intestinal microbiota and indices, including growth performance, serum immunity, sIgA in the ileum, and ileum morphological structure analyzed with a Spearman's correlation heatmap. **(A)** Correlation of microbiota with indices on starter phase (1–28 days). **(B)** Correlation of microbiota with indices on grower phase (29–56 days). The colors range from blue (negative correlation) to red (positive correlation). *represents *p* < 0.05 and ** represents *p* < 0.01.

Intestinal microbiota, which is the key factor to regulate the host intestinal health, can affect the barrier function of the intestinal tract and regulate intestinal immune response, and even systematic immune response (57). From our study, Spearman's correlation thermogram analysis showed that *Lactococcus* and *Leuconostoc* were a significant negative correlation with F/G (Figure 5). *Lactobacillus* were found to be still the dominant genus in poultry ileum and showed a significant positive correlation with poultry growth performance, in agreement with the previous results (58, 59). In the starter phase, the effects of three different oligosaccharide supplements on the intestinal microbiota of broilers were quite different. The proportion of *Lactobacillus* in the RFO group was dominant. Although the proportion of *Lactobacillus* in the COS group was almost the same as that in the RFO group, the ratio of *Candidatus_Arthromitus* was much higher than that of the RFO group. Both segmented filamentous bacteria SFB and Arthromitus filaments from arthropod guts were grouped under the provisional name “*Candidatus_Arthromitus*” as they share a striking similarity in morphology and close contact with the host gut wall (60). It is suggested that *Candidatus_Arthromitus* might be a cause of intestinal inflammation (61). These bacteria could adhere to the intestinal epithelium, especially the ileum. SFB played a key role in the maturation of gut innate and adaptive immune function in the postnatal gut. Notably, SFB can induce strong IgA responses (62, 63), and it may be one of the reasons for the difference in growth performance of the RFO group.

## Conclusions

In summary, both oligosaccharides and antibiotics can alter the intestinal microbiota, improve the antioxidant capacity, and then improve the growth performance of yellow-feathered broilers in our study. However, in terms of improved growth performance, RFO and COS showed superior effects over IMO. Additionally, the effect of RFO on growth performance was comparable to the use of antibiotics. IMO was greater than RFO and COS in improved intestinal mucosal barrier function. It is very potential to use oligosaccharides as an alternative to antibiotics to maintain growth performance and health in broilers.

## Data Availability Statement

The datasets generated for this study can be found in the NCBI- SAMN2589823.

## Ethics Statement

The animal study was reviewed and approved by Ethics of Animal Experiments of Hunan Agriculture University.

## Author Contributions

LC, YD, ZS, HZ, and XH designed the experiment and revised the manuscript. LC and YW carried out the animal trials, sample analysis, did some data analysis work, and edited the manuscript. LC, YW, ZS, and XH are responsible for the integrity of the work as a whole. All authors reviewed and approved the final manuscript.

## Funding

This study was supported by the Guangxi for Research Bases and Talents (Guike AD20238092), the China Agriculture Research System of MOF and MARA (CARS-41), and the Scientific and Technical Talents in Hunan Province (2020TJ-Q02).

## Conflict of Interest

FL was employed by Guangxi Fufeng Agriculture and Animal Husbandry Group Co., Ltd. The remaining authors declare that the research was conducted in the absence of any commercial or financial relationships that could be construed as a potential conflict of interest.

## Publisher's Note

All claims expressed in this article are solely those of the authors and do not necessarily represent those of their affiliated organizations, or those of the publisher, the editors and the reviewers. Any product that may be evaluated in this article, or claim that may be made by its manufacturer, is not guaranteed or endorsed by the publisher.
