# Supplementary material for: Effects of Different Oligosaccharides on Growth Performance and Intestinal Function in Broilers
Source: Front Vet Sci. 2022 Apr 1;9:852545. doi: 10.3389/fvets.2022.852545 (PMC9011052; doi:10.3389/fvets.2022.852545)
Supplement: Supplementary file 1 [file Data_Sheet_1.docx]

Table S1. Effects of dietary oligosaccharides on gut microbiota α diversity index of broilers in starter phase

| Item | CON | ANT | IMO | RFO | COS | P |
| --- | --- | --- | --- | --- | --- | --- |
| Chao1 | 493.90±111.43^a^ | 555.09±126.84^a^ | 622.11±4.49^a^ | 366.54±76.40^b^ | 225.09±28.89^c^ | ＜0.01 |
| Goods_coverage | 0.9941±0.0014^c^ | 0.9940±0.0010^c^ | 0.9941±0.0005^c^ | 0.9963±0.0004^b^ | 0.9976±0.0003^a^ | ＜0.01 |
| Observed_species | 314.64±88.05^bc^ | 404.53±127.31^ab^ | 500.27±25.00^a^ | 265.90±87.65^cd^ | 163.84±14.51^d^ | ＜0.01 |
| Shannon | 2.88±0.63^ab^ | 3.14±0.92^ab^ | 3.54±0.32^a^ | 2.41±0.66^bc^ | 1.79±0.80^c^ | 0.023 |

Note: Figures with different superscripts within the same column are significantly different (P < 0.05), n=5. Each mean represents 5 replicates per treatment, with 1 layers per replicate.

Table S2. Effects of dietary oligosaccharides on gut microbiota α diversity index of broilers in grower phase

| Item | CON | ANT | IMO | RFO | COS | P |
| --- | --- | --- | --- | --- | --- | --- |
| Chao1 | 277.94±38.42^b^ | 282.81±87.57^b^ | 279.73±76.50^b^ | 491.99±48.66^a^ | 359.98±75.26^b^ | ＜0.01 |
| Goods_coverage | 0.9971±0.0003^a^ | 0.9971±0.0014^a^ | 0.9971±0.0006^a^ | 0.9947±0.0007^b^ | 0.9958±0.0007^b^ | ＜0.01 |
| Observed_species | 201.34±25.57^b^ | 207.76±72.73^b^ | 204.76±71.78^b^ | 349.80±37.71^a^ | 225.20±73.27^b^ | 0.003 |
| Shannon | 2.45±0.79 | 2.69±0.55 | 2.66±0.79 | 3.15±0.79 | 2.46±0.65 | 0.549 |

Note: Figures with different superscripts within the same column are significantly different (P < 0.05), n=5. Each mean represents 5 replicates per treatment, with 1 layers per replicate.

Table S3. Effects of dietary oligosaccharides on gut microbiota of broilers in starter phase (phylum level)

| Item | CON | ANT | IMO | RFO | COS | P |
| --- | --- | --- | --- | --- | --- | --- |
| *p__Acidobacteria* | 0.0009±0.00147 | 0.0001±0.00007 | 0.0001±0.00009 | 0.0000±0.0000 | 0.0000±0.0000 | 0.275 |
| *p__Spirochaetae* | 0.0017±0.00185^c^ | 0.0120±0.00884^b^ | 0.0189±0.00384^a^ | 0.0001±0.00008^c^ | 0.0000±0.0000^c^ | ＜0.01 |
| *p__Proteobacteria* | 0.0063±0.00412 | 0.0085±0.00440 | 0.0381±0.5290 | 0.0117±0.00785 | 0.0018±0.00057 | 0.145 |
| *p__Cyanobacteria* | 0.0487±0.05190 | 0.0110±0.00646 | 0.0084±0.01087 | 0.0034±0.00341 | 0.0015±0.00175 | 0.058 |
| *p__Planctomycetes* | 0.0001±0.00013^bc^ | 0.0002±0.00026^ab^ | 0.0003±0.00012^a^ | 0.0000±0.0000^c^ | 0.0000±0.0000^c^ | 0.009 |
| *p__Bacteroidetes* | 0.0123±0.00741^bc^ | 0.0363±0.02154^ab^ | 0.0566±0.01672^a^ | 0.0226±0.03049^bc^ | 0.0041±0.00162^c^ | 0.01 |
| *p__Firmicutes* | 0.7291±0.19621^b^ | 0.9007±0.04055^a^ | 0.8532±0.07123^b^ | 0.9562±0.04104^a^ | 0.9641±0.05372^a^ | 0.016 |
| *p__Actinobacteria* | 0.1977±0.18805^a^ | 0.0226±0.01520^b^ | 0.0059±0.00268^b^ | 0.0016±0.0006^b^ | 0.0005±0.00014^b^ | 0.015 |
| *p__Verrucomicrobia* | 0.0003±0.00020 | 0.0013±0.00124 | 0.0057±0.00732 | 0.0001±0.00006 | 0.0253±0.05328 | 0.518 |
| *p__Euryarchaeota* | 0.0005±0.00063^b^ | 0.0006±0.00067^b^ | 0.0017±0.00013^a^ | 0.0001±0.00023^b^ | 0.0000±0.0000^b^ | 0.001 |
| *p__Tenericutes* | 0.0018±0.00085^b^ | 0.0051±0.00337^b^ | 0.0088±0.00305^a^ | 0.0037±0.00246^b^ | 0.0027±0.00115^b^ | 0.005 |

Note: Figures with different superscripts within the same column are significantly different (P < 0.05), n=5. Each mean represents 5 replicates per treatment, with 1 layers per replicate.

Table S4. Effects of dietary oligosaccharides on gut microbiota of broilers in grower phase (phylum level)

| Item | CON | ANT | IMO | RFO | COS | P |
| --- | --- | --- | --- | --- | --- | --- |
| *p__Bacteroidetes* | 0.0162±0.00629 | 0.0093±0.00675 | 0.0315±0.02765 | 0.1447±0.23849 | 0.0081±0.01000 | 0.071 |
| *p__Firmicutes* | 0.9630±0.02083 | 0.7328±0.24930 | 0.9277±0.05374 | 0.5716±0.30774 | 0.7958±0.28927 | 0.431 |
| *p__Actinobacteria* | 0.0018±0.00183 | 0.1224±0.23086 | 0.0035±0.00211 | 0.0103±0.01019 | 0.0056±0.00562 | 0.367 |
| *p__Proteobacteria* | 0.0085±0.00483 | 0.0838±0.10733 | 0.0303±0.03288 | 0.0170±0.01725 | 0.0206±0.03119 | 0.121 |
| *p__Cyanobacteria* | 0.0082±0.01592 | 0.0081±0.00739 | 0.0036±0.00310 | 0.2444±0.30436 | 0.1656±0.24079 | 0.431 |
| *p__Tenericutes* | 0.0022±0.00020 | 0.0013±0.00106 | 0.0026±0.00228 | 0.0072±0.00890 | 0.0016±0.00104 | 0.20 |

Note: Figures with different superscripts within the same column are significantly different (P < 0.05), n=5. Each mean represents 5 replicates per treatment, with 1 layers per replicate.

Table S5. Effects of dietary oligosaccharides on gut microbiota of broilers in starter phase (species level)

| Item | CON | ANT | IMO | RFO | COS | P |
| --- | --- | --- | --- | --- | --- | --- |
| *g_Lactobacillus* | 0.5428±0.21712^b^ | 0.2248±0.08288^c^ | 0.5493±0.16486^b^ | 0.8377±0.08395^a^ | 0.8461±0.14204^a^ | ＜0.01 |
| *g_Candidatus_Arthromitus* | 0.0472±0.03302^b^ | 0.4829±0.17805^a^ | 0.1485±0.22098^b^ | 0.0036±0.00486^b^ | 0.0513±0.07235^b^ | ＜0.01 |
| *g_Faecalibacterium* | 0.0063±0.00239^bc^ | 0.0248±0.01748^a^ | 0.0178±0.00504^ab^ | 0.0057±0.00558^bc^ | 0.0033±0.00219^c^ | 0.005 |
| *g_Bacteroides* | 0.0053±0.00245^b^ | 0.0115±0.00719^a^ | 0.0253±0.016129^b^ | 0.0046±0.00190^b^ | 0.0018±0.00045^b^ | 0.001 |
| *g_Romboutsia* | 0.0032±0.00329 | 0.0025±0.00284 | 0.0053±0.00477 | 0.0019±0.00154 | 0.0010±0.00082 | 0.315 |
| *g_Escherichia_Shigella* | 0.0020±0.00053 | 0.0048±0.00387 | 0.0347±0.05131 | 0.0037±0.00232 | 0.0008±0.00050 | 0.119 |
| *g_Ruminococcaceae_UCG_014* | 0.0011±0.00033^c^ | 0.0029±0.00087^ab^ | 0.0052±0.00216^a^ | 0.0032±0.00306^ab^ | 0.0012±0.00048^c^ | 0.025 |
| *g_Anaerotruncus* | 0.0013±0.00050 | 0.0021±0.00066 | 0.0026±0.00091 | 0.0023±0.00125 | 0.0013±0.00066 | 0.121 |
| *g_Lachnoclostridium* | 0.0008±0.00017^b^ | 0.0013±0.00045^b^ | 0.0029±0.00058^a^ | 0.0027±0.00129^a^ | 0.0012±.00047^b^ | 0.002 |
| *g_Ruminococcustorquesgroup* | 0.0006±0.00016 | 0.0013±0.00074 | 0.0019±0.00060 | 0.0018±0.00119 | 0.0007±0.00057 | 0.061 |
| *g_Rikenella* | 0.0013±0.00112 | 0.0037±0.00348 | 0.0026±0.00131 | 0.0021±0.00074 | 0.0013±.00070 | 0.248 |
| *g_Treponema_2* | 0.0016±0.00176^c^ | 0.0116±0.00853^b^ | 0.0184±0.00380^a^ | 0.0001±0.00008^c^ | 0.0000±0.0000^c^ | ＜0.01 |

Note: Figures with different superscripts within the same column are significantly different (P < 0.05), n=5. Each mean represents 5 replicates per treatment, with 1 layers per replicate.

Table S6. Effects of dietary oligosaccharides on gut microbiota of broilers in grower phase (species level)

| Item | CON | ANT | IMO | RFO | COS | P |
| --- | --- | --- | --- | --- | --- | --- |
| *g_Lactobacillus* | 0.8686±0.01477 | 0.5450±0.34908 | 0.8359±0.11675 | 0.4534±0.35340 | 0.6482±0.25994 | 0.079 |
| *g_Faecalibacterium* | 0.0128±0.00608 | 0.0031±0.00239 | 0.0042±0.00484 | 0.0131±0.01329 | 0.0043±0.00271 | 0.073 |
| *g_Escherichia_Shigella* | 0.0070±0.00491 | 0.0064±0.00699 | 0.0163±0.02779 | 0.0013±0.0059 | 0.0021±0.00072 | 0.409 |
| *g_Bacteroides* | 0.0056±0.01306 | 0.0056±0.00270 | 0.0033±0.00196 | 0.0059±0.00721 | 0.0177±0.02239 | 0.264 |
| *g_Rikenella* | 0.0025±0.00115 | 0.0009±0.00087 | 0.0045±0.00656 | 0.0097±0.01439 | 0.0009±0.00110 | 0.297 |
| *g_Anaerotruncus* | 0.0016±0.00087 | 0.0006±0.00036 | 0.0018±0.00202 | 0.0018±0.00186 | 0.0007±0.00040 | 0.405 |
| *g_Ruminococcaceae_UCG_014* | 0.0024±0.00094 | 0.0011±0.00059 | 0.0023±0.00223 | 0.0038±0.00430 | 0.0018±0.00170 | 0.151 |
| *g_Ruminococcustorquesgroup* | 0.0016±0.00074 | 0.0014±0.00079 | 0.0029±0.00369 | 0.0073±0.00776 | 0.0010±0.00116 | 0.108 |
| *g_Lachnoclostridium* | 0.0014±0.00065 | 0.0006±0.00030 | 0.0019±0.00210 | 0.0026±0.00315 | 0.0008±0.00060 | 0.376 |
| *g_Alistipes* | 0.0011±0.00046 | 0.0005±0.00055 | 0.0030±0.00292 | 0.0085±0.01544 | 0.0004±0.00091 | 0.357 |
| *g_Butyricicoccus* | 0.0012±0.00059 | 0.0005±0.00018 | 0.0030±0.00397 | 0.0011±0.00117 | 0.0004±0.00030 | 0.222 |
| *g_Rothia* | 0.0008±0.00120 | 0.0782±0.17058 | 0.0015±0.00088 | 0.0049±0.00923 | 0.0007±0.00073 | 0.432 |
| *g_Romboutsia* | 0.0031±0.00505 | 0.0013±0.00093 | 0.0072±0.01361 | 0.0020±0.00179 | 0.0893±0.13876 | 0.15 |

Note: Figures with different superscripts within the same column are significantly different (P < 0.05), n=5. Each mean represents 5 replicates per treatment, with 1 layers per replicate.


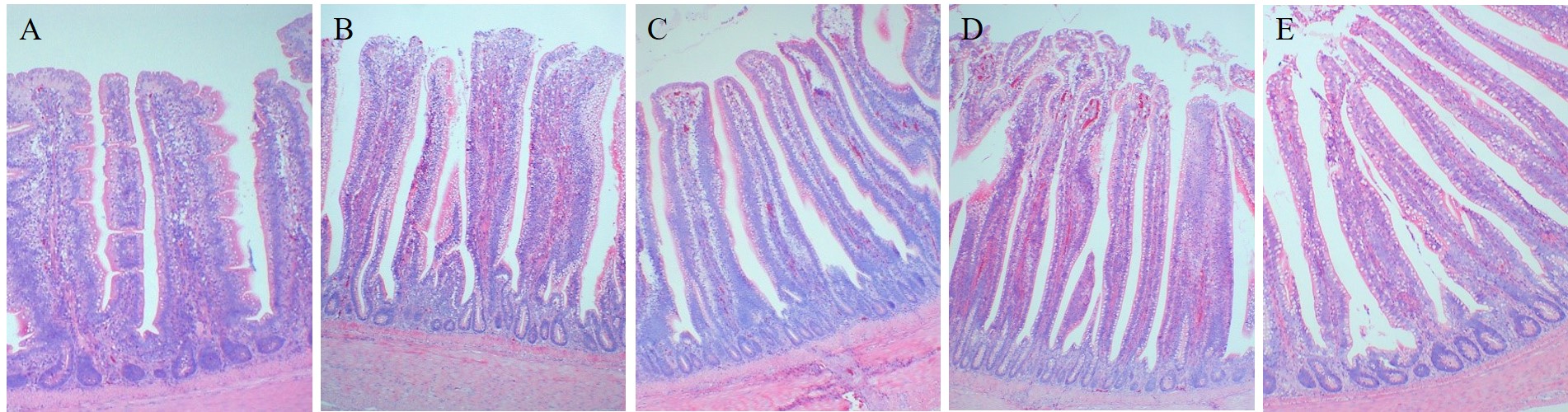


Figure S1 Effects of dietary oligosaccharides on Intestinal Histomorphology of broilers（40×）(A) CON, control group; (B) ANT, ANT group; (C) IMO, IMO group; (D) RFO, RFO group; (E) COS, COS group.
